# Supplementary material for: Follicular Fluid Amino Acid Alterations in Endometriosis: Evidence for Oxidative Stress and Metabolic Dysregulation
Source: Biomedicines. 2025 Oct 27;13(11):2634. doi: 10.3390/biomedicines13112634 (PMC12650251; doi:10.3390/biomedicines13112634)
Supplement: Supplementary file 1 [file biomedicines-13-02634-s001.zip › biomedicines-3861119-supplementary.docx]

1. Amino Acid Analysis
   1. Reagents

The following chemicals were used for amino acid analysis: 3-mercaptopropionic acid (≥99.0%, HPLC grade), ortho-phthalaldehyde (≥99%, HPLC grade), and 9-fluorenylmethyloxycarbonyl chloride (FMOC chloride) (≥99.0%, HPLC grade), all obtained from Merck KGaA, Darmstadt, Germany. Additional reagents included acetonitrile (≥99.9%, HPLC gradient grade), methanol (≥99.8%, HPLC grade), and HPLC gradient-grade water, all supplied by Fisher Chemical, Pittsburgh, Pennsylvania, USA. A 20 mM phosphate buffer (pH 6.2, KH2PO4: Fisher Chemical, KOH: Lach-Ner Ltd., Czech Republic) was used for sample preparation. The mobile phase for UHPLC elution consisted of a mixture of 400 mL acetonitrile, 450 mL methanol, and 150 mL water. L-Norvaline (Merck KGaA, Darmstadt, Germany) was used as an internal standard.

- 1. Sample Preparation for UHPLC Measurement

Quantitative amino acid analysis of follicular fluid samples was conducted after protein precipitation, fluorescence derivatization, and UHPLC chromatography. The analyses were performed using a Shimadzu Nexera X2 UHPLC System equipped with a fluorescence detector (RF-20A XS, Shimadzu Europa GmbH, Duisburg, Germany) and an internal standard (250 µmol/L L-Norvaline). For sample preparation, 300 µL of ice-cold acetonitrile was added to 200 µL of follicular fluid sample, followed by vortex mixing and centrifugation at 6100 × g for 4 minutes (ScanSpeed Mini, Labogene, Allerød, Denmark). After centrifugation, 300 µL of the supernatant was mixed with 600 µL of phosphate buffer. The mixture was filtered using a Millex® GV 4 mm Durapore PVDF 0.22 µm filter (Merck KGaA, Darmstadt, Germany) and placed into the autosampler module (SIL-30AC Autosampler) for analysis.

- 1. Derivatization

Amino acid derivatization was performed using 3-mercaptopropionic acid (MPA) and ortho-phthalaldehyde (OPA). For proline, 9-fluorenylmethyloxycarbonyl chloride (FMOC) was used instead. The derivatization process involved mixing 7.5 µL of sample, 45 µL of MPA, 22 µL of OPA and 3 µL of 250 µmol/L L-Norvaline. The mixture was incubated for 1 minute, followed by the addition of 10 µL of FMOC reagent and further incubation for 2 minutes. Finally, 5 µL of the derivatized sample was injected into the UHPLC system.

- 1. UHPLC Method Parameters

Each 5 µL sample aliquot was injected into the UHPLC system. Separation was achieved using a reverse-phase Kinetex 2.6 μm EVO C18 100Å column (100 × 3.0 mm, Phenomenex, Torrance, CA, USA). The mobile phase consisted of 20 mM phosphate buffer (A) and 40:45:15 acetonitrile:methanol:water solution (B). The flow rate was set at 1.3 mL/min, with the column temperature maintained at 27°C. The total running time for each sample was 15.1 minutes. Amino acids (except proline) were detected using a fluorescence detector (RF-20A XS, Shimadzu) with an excitation wavelength of 350 nm and an emission wavelength of 450 nm. Proline was detected separately at an excitation wavelength of 266 nm and an emission wavelength of 305 nm. Data acquisition and analysis were performed using Shimadzu LabSolutions 5.97 SP1 software. Each amino acid was identified based on its retention time (RT), and concentrations were calculated using the area under the curve (AUC) of the internal standard. All samples were measured in duplicate, with the final concentration determined as the average of both measurements.
